# Associations between locomotive and non-locomotive physical activity and physical performance in older community-dwelling females with and without locomotive syndrome: a cross-sectional study

**DOI:** 10.1186/s40101-021-00268-8

**Published:** 2021-11-04

**Authors:** Tomohiro Nishimura, Atsushi Hagio, Kanako Hamaguchi, Toshiyuki Kurihara, Motoyuki Iemitsu, Kiyoshi Sanada

**Affiliations:** 1 Faculty of Sport and Health Science, Ritsumeikan University, 1-1-1 Noji Higashi, Kusatsu, Shiga, 525-8577 Japan; 2 Osaka College of Rehabilitation, Osaka, 530-0043 Japan; 3 Department of Public Health Care and Welfare of Yawata City, Kyoto, 614-8501 Japan; 4 Research Organization of Science and Technology, Ritsumeikan University, Shiga, 525-8577 Japan

**Keywords:** Locomotive syndrome, Moderate to vigorous physical activity, Walking speed, Older community-dwelling females, Locomotive physical activity, Daily step counts

## Abstract

**Background:**

Locomotive syndrome (LS) is a condition of reduced mobility due to a disorder of the locomotive system. Increasing moderate to vigorous physical activity (MVPA) has been recommended to prevent LS. However, to increase daily MVPA is difficult for older people with LS. The MVPA consists of not only locomotive activities such as walking but also non-locomotive activities such as household activities. The aim of this study was to examine the associations between locomotive/non-locomotive MVPA and physical performance in older females with and without LS.

**Methods:**

Participants of this cross-sectional study were 143 older community-dwelling Japanese females. The participants were divided into two groups based on the results of the stand-up test: the normal group (NL) (*n* = 86) and the LS group (*n* = 57). Both the locomotive and non-locomotive PA seperately measured with its intensity. The intensity of physical activity (PA) was calculated as METs and classified as sedentary behavior (SB 1–1.5 metabolic equivalent tasks (METs)), low-intensity physical activity (LPA 1.6–2.9 METs), and MVPA (≥ 3 METs). For example, locomotive LPA is slow walking speed of 54 m/min, and locomotive MVPA is walking speed of 67 m/min. While non-locomotive LPA is office work and cooking, non-locomotive MVPA is housecleaning. Physical function was evaluated by handgrip strength, walking speed, and 2-step test.

**Results:**

Walking speed, hand-grip strength, 2-step test, daily step counts, and all PA measurements were not significantly different between two groups. In the LS, locomotive MVPA (*r* = 0.293, *p* < 0.05) and total MVPA (*r* = 0.299, *p* < 0.05) was significantly correlated with walking speed, but not in the NL.

**Conclusions:**

Walking speed was positively correlated with locomotive MVPA and total MVPA in the LS group, but not in NL group. This result suggests that slow walking speed in older people with LS occur in connection with lower locomotive MVPA and total MVPA.

## Background

Locomotive syndrome (LS) is a condition of reduced mobility due to a disorder of the locomotive system, including bones, joints, muscles, and nerves [1, 2], which proposed by the Japanese Orthopedics Association (JOA) in 2007 [1]. This concept had established by the evidence that the most older people, who were required care services, have locomotive problems [1, 2]. Some previous studies indicated that a decline in performance of activities of daily living (ADL), a low degree of independence, and frailty in older people are primarily caused by LS [3, 4]. Kobayashi et al. [5] conducted a 5-year cohort study to investigate the risk of LS in 113 Japanese community-dwelling older individuals using physical functional tests proposed by the JOA (i.e., the 2-step test, stand-up test, and the 25-question Geriatric Locomotive Function Scale (GLFS-25)), and reported that screening for LS risk is useful for predicting future motor dysfunction in middle-aged and older people.

Increasing moderate to vigorous physical activity (MVPA) has been recommended to prevent LS. The “Active Guide 2013”, a guideline for health promotion by the Japanese Ministry of Health, Labour and Welfare (MHLW) [6], proposed a plan to increase daily physical activity (PA), especially moderate to vigorous intensity physical activity, by performing 23 metabolic equivalent tasks (METs)-h/week in order to prevent lifestyle-related diseases and dysfunction [6]. Meanwhile, the World Health Organization issued the “Global Recommendations on Physical Activity for Health” in 2010, stating that 30 min of MVPA per day can effectively improve muscular fitness and functional health [7]. With a focus on extending healthy life span, MHLW proposed a plan “Healthy Japan 21”, which recommends walking 8500 steps a day in the case of 18–64 age, and walking 6000 steps a day in the case of 65 age and over (equivalent to 60 min of MVPA per day including walking and ADL) in order to increase PA [8].

For the patients with LS who have difficulties in locomotion, the intervention to increase PA by increasing step counts are not feasible. Thus, an alternative intervention is needed for the patients with LS. Non-locomotive PA which was except for walking and running, such as household work, has been supposed to contribute for increasing the MVPA [9, 10]. Oshima et al. [9] introduced the method to distinguish non-locomotive MVPA from locomotive MVPA based on triaxial accelerometer data, and suggested that non-locomotive MVPA should be considered when assessing MVPA [9]. Tanaka et al. [10] measured PA in the same manner as Oshima et al.’s method and determined a duration of non-locomotive MVPA (including performing yard work, agricultural tasks, and household activities, in community-dwelling Japanese people), and showed that non-locomotive MVPA occupied more than half of total MVPA in community-dwelling Japanese people (571 females and 315 males; age range, 18–92 years) [10]. They also reported that females in all age groups spent more time on non-locomotive MVPA than males [10]. Thus, it is important to evaluate both locomotive and non-locomotive MVPA when estimating the duration of MVPA required to prevent and/or improve LS. However, to the best of our knowledge, no study has compared the duration of locomotive and non-locomotive MVPA between older females with LS and those without LS.

A walking speed is impaired with LS [11], since it is directly related to locomotive dysfunction. In older community-dwelling Japanese females, a positive relationship between the duration of MVPA and walking speed was reported in a previous paper [12]. In that study, MVPA of locomotive (e.g., walking and running) and non-locomotive (e.g., household activities) activities were not separated, however as aforementioned, a large proportion of MVPA in older females consists of non-locomotive MVPA [10]. Thus, the relationship between walking speed and non-locomotive MVPA might differ between older individuals with LS and those without LS. We hypothesized that the walking speed is strongly influenced by the duration of non-locomotive MVPA, especially in the older people with LS. To this end, the present cross-sectional study aimed to (1) investigate whether the duration of locomotive MVPA and non-locomotive MVPA are longer or shorter in the older females with LS compared to those without LS, and (2) examine the association between locomotive/non-locomotive MVPA and walking ability in older females with and without LS.

## Methods

### Study design and participants

This study was a cross-sectional study conducted to investigate the association between non-locomotive MVPA and walking speed in older females with LS. In the present study, we recruited the participants by leaflets and a community newsletter of health events in Yawata City, Kyoto, and health physical fitness test by advertisement of the newspaper in Kusatsu City, Shiga, Japan, in 2019. One hundred forty-three community-dwelling Japanese older males and females (57 and 86, respectively) participated from Yawata City, and 110 community-dwelling Japanese older females participated from Kusatsu City. Two hundred fifty-three older community-dwelling Japanese participated in the briefing session. The exclusion criteria in the present study were as follows:(1) males, (2) less than 60 years old, (3) participants with adrenal gland disease, heart disease, severe liver dysfunction, cirrhosis, severe renal dysfunction, and dementia, (4) participants who are pregnant or suspected of being pregnant, (5) participants who are outpatient for orthopedics or have limited exercise, and (6) participant who could not live without assistive and nursing care. In the present study, 57 participants were excluded by above criteria, and 53 participants were excluded after the data collection because of incomplete data of a triaxial accelerometer (the details were described later). Finally, 143 older community-dwelling Japanese females aged ≥ 60 years (mean age, 68.2 ± 4.8 years) participated. All the participants were informed of the benefits and risks of the study and provided written informed consent when they voluntarily participated in this study. This study was approved by the Ethics Committee of Ritsumeikan University (BKC-IRB-2018-074).

### Measurements of physical characteristics and physical function

Anthropometric parameters, including body weight, height, body mass index (BMI), appendicular muscle mass (AMM), and skeletal muscle index (SMI) were measured. BMI was calculated as body weight (kg) divided by height squared (m^2^). AMM was measured by bioelectrical impedance analysis (BIA) using the Tanita BC545N scale (TANITA Corporation, Japan). AMM was derived as the sum of the lean soft tissue mass of the arms and legs [13]. SMI was calculated as AMM (kg) divided by height squared (m^2^).

Handgrip strength, walking speed, and 2-step test were measured as the physical function. Handgrip strength was measured with a hand dynamometer (TKK 5401, Takei Scientific Instruments Co., Ltd., Japan). The greater value of measurements from two trials for each hand was recorded. To measure the usual walking speed, the participants were asked to walk 10 meters straight at their usual speed, and the walking speed over the middle six meters was calculated. Measurements of walking speed were performed twice, and the faster speed was recorded. In the 2-step test, the participants were asked to stand with the toes of both feet behind the starting line, take two steps as far as possible, and stand again with both feet aligned. The length of two steps was measured from the starting line to the tips of the toes [14]. The test was performed twice, and the greater value normalized by body height was recorded.

### Classification of LS and assessment of walking ability

In general, it is recommended to evaluate LS risk by the combination of the 2-step test, stand-up test, and GLFS-25 [14–16]. In the present study, the participants were divided into two groups based on the results of the stand-up test: the NL group (*n* = 86) and the LS group (*n* = 57). In short, if the participant was unable to stand up on one leg (right or left) from a height of 40 cm chair, then she was determined to have LS [17]. In the present study, the 2-step test was used as an index of balance and walking ability, and GLFS-5 was used as an index of activities of daily living (ADL). GLFS-5 is commonly used as a short version of GLFS-25 [16] in the LS evaluation, since the former is used to measure stride length, which is known to reflect walking ability as well as muscle strength, balance, and flexibility of the lower limbs [14], and the latter is associated with a decline in mobility in ADL performance [16].

Walking ability is generally assessed by daily step counts, usual walking speed, and the 2-step test [14, 18, 19]. As mentioned above, since the 2-step test is closely related to the LS risk tests, we assessed walking ability based on daily step counts and usual walking speed.

### Assessment of ADL performance

ADL performance was assessed by using the Tokyo Metropolitan Institute of Gerontology, Index of Competence (TMIG-IC), a multidimensional 13-item scale, and the GLFS-5 [20, 21]. In TMIG-IC, the participants were asked to respond to each item with either a “yes” (able to do; 1 point) or “no” (unable to do; 0 points), for a maximum score of 13 points. Higher scores reflect a higher level of functional ability. The participants with a total TMIG-IC score ≥ 11 were considered to be able to live independently in the community, and lower than 11 were considered to have decreased higher-level functional capability [20, 21]. Seichi et al. found that GLFS-5 can be applied as a rapid self-check tool for LS. The GLFS-5 is graded on a 5-point scale, from no impairment (0 points) to severe impairment (4 points), and scores for each item are added to obtain a total score (minimum = 0, maximum = 20). Higher GLFS-5 scores are associated with greater impairment of locomotive function. The validity of this scale has been assessed, and a cut-off point of six has been determined to have the highest sensitivity and specificity for detecting disability resulting from LS [16].

### Assessment of physical activity and step counts

PA and daily step counts (steps/day) were recorded using a triaxial accelerometer (Active style Pro, Omron Healthcare Co., Ltd.: HJA-350IT). The participants were asked to wear a triaxial accelerometer around their waist during the daytime for 3 weeks. PA data were adopted if accelerometer data were successfully collected for 7 days, including weekdays and weekends, during the 3-week period. Daily data collection was considered successful if at least 600 min of wearing time per day could be confirmed [22, 23]. The data of the participants who could not achieve the 7 days of successful data collection were excluded from this study.

Active style Pro can classify PA into locomotive and non-locomotive PA in the same manner as the previous studies [9, 10]. In short, it can distinguish PA by the algorism based on the change in acceleration waveform amplitude and duration, and the inclination of the upper body of PA [9, 10]. When there is no change in the inclination of the upper body, it is classified as a locomotive PA, and when there is a change in inclination, it is classified as a non-locomotive PA. The intensity of PA was calculated as METs and classified as sedentary behavior (SB 1–1.5 METs), low-intensity physical activity (LPA 1.6–2.9 METs), and MVPA (≥ 3 METs). For example, locomotive LPA is slow walking speed of 54 m/min, and locomotive MVPA is walking speed of 67 m/min. While non-locomotive LPA is office work and cooking, non-locomotive MVPA is housecleaning and gardening.

### Statistical analysis

All measurements and calculated values are expressed as mean ± standard deviation (SD). Mean values of anthropometric and physical function measurements, walking ability, ADL performance, and PA were firstly checked the normal distribution using the Shapiro-Wilk normality test, and Measurement values of age, weight, AMM, walking speed, 2-step test value, GLFS-5, TMIG-IC, daily step counts, sedentary behavior, locomotive LPA, locomotive MVPA, non-locomotive MVPA, MVPA, and total locomotive PA were confirmed as the non-normal distribution, and the data was transformed in a log transformation. In the comparing the two groups, a statistical analysis was performed with the unpaired *t* test. There was a significant difference in age and weight between the LS and NL groups so that statistical analysis was performed with analysis of covariance (ANCOVA) adjusted for age and weight between two groups. In the relationships between walking ability and MVPA, statistical analysis was performed with partial correlation coefficient, age and body weight were used as a covariance with walking speed and MVPA. The alpha level for testing significance was set at *P* < 0.05. All statistical analyses were performed using SPSS Statistical Software, Version 25 (SPSS, Inc., Tokyo, Japan).

## Results

Anthropometric measurements and physical function were summarized in Table 1. Age and body weight were significantly higher and heavier in the LS group compared to the NL group. All measurements except for age and weight were analyzed by ANCOVA adjusted for age and weight because these were exhibited significant differences in the two groups with the unpaired *t* test. No significant differences were observed in height, BMI, AMM, and SMI between the two groups. In the physical function, there was a significant difference in the 2-step test by the unpaired *t* test, but the 2-step test and walking speed did not significantly differ between the two groups by ANCOVA adjusted for age and weight. In ADL performance, The GLFS-5 score was significantly higher in the LS group compared to the NL group, whereas TMIG-IC scores showed no significant difference between the two groups with the unpaired *t* test. All measurements except the GLFS-5 score did not significantly differ between the two groups of ANCOVA adjusted for age and weight. The GLFS-5 score showed significant difference between the two groups of ANCOVA adjusted for age and weight (Table 1). Table 2 showed the daily step counts and PA. Daily step counts and all PA measured by using a triaxial accelerometer were significantly different in daily step counts, total locomotive PA, and locomotive MVPA by the unpaired t-test, but not significantly different between the two groups by ANCOVA adjusted for age and weight.

**Table 1:** Physical characteristics and physical function of the subjects

	Total (*n* = 143)	NL (*n* = 86)	LS (*n* = 57)	*p* value	
	Mean SD	Mean SD	Mean SD	*t* test	ANCOVA
Age (years)	68.2 ± 4.8	67.3 ± 4.8	69.6 ± 4.5	0.005*	-
Height (cm)	155.0 ± 5.6	154.5 ± 5.6	155.7 ± 5.5	0.216	0.398
Weight (kg)	53.3 ± 7.7	52.0 ± 6.9	55.2 ± 8.4	0.018*	-
BMI (kg/m^2^)	22.2 ± 2.8	21.8 ± 2.7	22.7 ± 2.9	0.055	0.398
AMM (kg)	16.0 ± 1.6	15.8 ± 1.5	16.3 ± 1.8	0.108	0.394
SMI (kg/m^2^)	6.65 ± 0.59	6.62 ± 0.55	6.71 ± 0.65	0.414	0.127
Handgrip strength (kg)	24.3 ± 3.3	24.4 ± 3.5	24.0 ± 3.1	0.540	0.163
Walking speed (m/s)	1.52 ± 0.18	1.54 ± 0.16	1.49 ± 0.20	0.076	0.135
2-step test value (cm/cm)	1.36 ± 0.15	1.39 ± 0.14	1.31 ± 0.15	0.002*	0.061
GLFS-5 (score)	1.55 ± 2.25	1.07 ± 1.49	2.26 ± 2.94	0.009*	0.046*
TMIG-IC (score)	12.7 ± 0.7	12.7 ± 0.8	12.6 ± 0.7	0.968	0.664

Mean ± SD, *NL* normal group, *LS* locomotive syndrome group, *BMI* body mass index, *AMM* appendicular muscle mass, *SMI* skeletal muscle mass index, *GLFS-5* 5-question Geriatric Locomotive Function Scale, *TMIG-IC* Tokyo Metropolitan Institute of Gerontology Index of Competence, *SD* standard deviation, *p* value, the unpaired *t* test, *ANCOVA* analysis of covariance, covariate: age and weight

*p < 0.05

**Table 2:** Daily step counts and physical activities

	Total (*n* = 143)	NL (*n* = 86)	LS (*n* = 57)	*p* value	
	Mean SD	Mean SD	Mean SD	*t* test	ANCOVA
Daily step counts (steps/day)	6608 ± 2444	6994 ± 2418	6025 ± 2409	0.013*	0.144
SB (h)	7.0 ± 1.4	7.0 ± 1.5	7.1 ± 1.2	0.467	0.586
Total PA (METs·h)	18.4 ± 3.6	18.9 ± 3.7	17.6 ± 3.2	0.056	0.256
LocomotivePA (METs·h)	3.3 ± 1.5	3.5 ± 1.6	3.1 ± 1.3	0.047*	0.377
Non-locomotivePA (METs·h)	15.0 ± 3.2	15.4 ± 3.4	14.5 ± 3.0	0.188	0.330
LPA (METs·h)	14.6 ± 2.8	14.9 ± 2.8	14.2 ± 2.8	0.178	0.261
LocomotivePA (METs·h)	1.5 ± 0.6	1.5 ± 0.6	1.5 ± 0.6	0.836	0.931
Non-locomotivePA (METs·h)	13.2 ± 2.7	13.4 ± 2.7	12.8 ± 2.7	0.188	0.233
MVPA (METs·h)	3.7 ± 1.8	4.0 ± 2.0	3.4 ± 1.6	0.064	0.561
LocomotivePA (METs·h)	1.9 ± 1.2	2.0 ± 1.2	1.6 ± 1.1	0.013*	0.213
Non-locomotivePA (METs·h)	1.9 ± 1.1	2.0 ± 1.1	1.8 ± 1.0	0.651	0.830

Mean ± SD, *NL* normal group, *LS* locomotive syndrome group, *SB* sedentary behavior, *LPA* low-intensity physical activity, *MVPA* moderate to vigorous intensity physical activity, *PA* physical activity, *SD* standard deviation, *h* hour, *p* value, the unpaired *t* test, *ANCOVA* analysis of covariance, covariate: age and weight

*p < 0.05

**Fig. 1:**
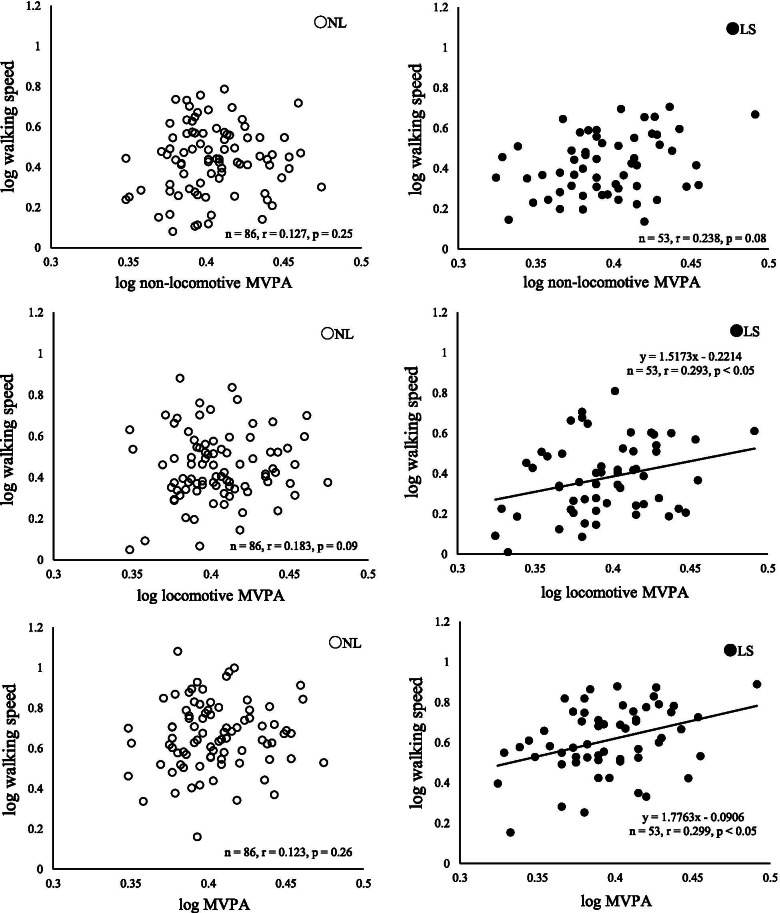
Relationships between walking speed and MVPA. Relationships between walking speed and non-locomotive MVPA (top), locomotive MVPA (middle), and total MVPA (bottom) in the LS group and NL group. Open circle: NL group, closed circle: LS group. Partial correlation coefficients adjusted for age and body weight were calculated

**Fig. 2:**
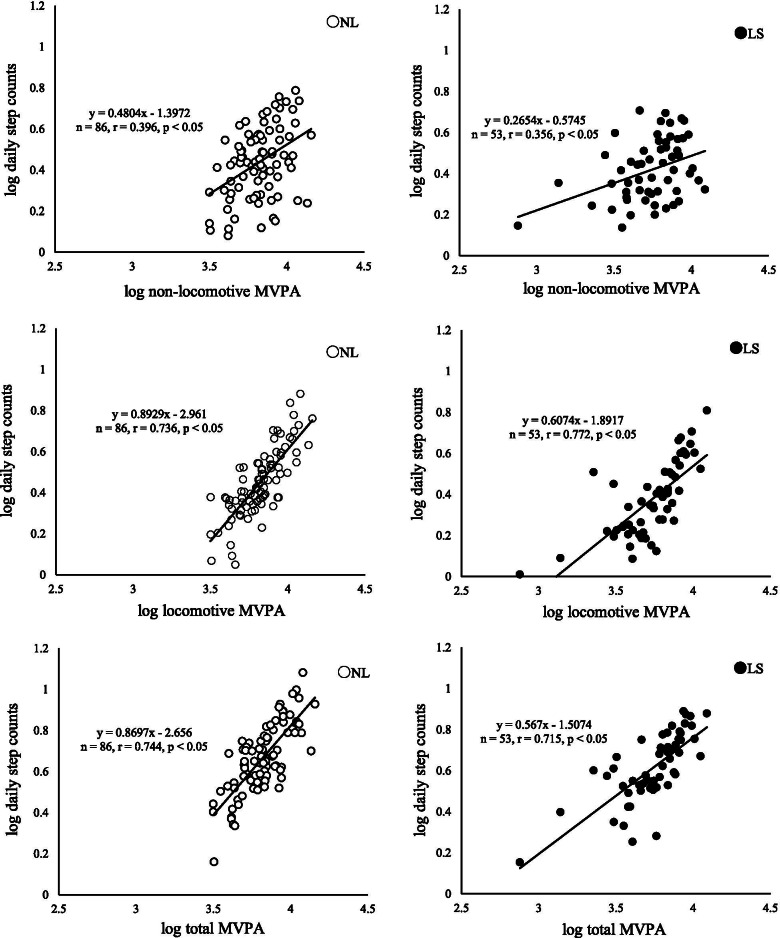
Relationships between daily step counts and MVPA. Relationships between daily step counts and non-locomotive MVPA (top), locomotive MVPA (middle), and MVPA (bottom) in the LS group and NL group. Open circle: NL group, closed circle: LS group. Partial correlation coefficients adjusted for age and weight were calculated

Walking speed was significantly correlated with locomotive MVPA (*r* = 0.293, *p* < 0.05) and total MVPA (*r* = 0.299, *p* < 0.05) in the LS group, but not in the NL group (Fig. 1). No significant correlations were observed between non-locomotive MVPA and walking speed in both groups. In walking ability except for walking speed, the 2-step test in the LS group was significantly correlated with locomotive MVPA (*r* = 0.282, *p* < 0.05), but not significantly with non-locomotive MVPA, whereas the 2-step test in the NL group was significantly correlated with non-locomotive MVPA (*r* = 0.216, *p* < 0.05), but not significantly with locomotive MVPA. Significant correlations were observed between daily step counts and locomotive MVPA, between daily step counts and non-locomotive MVPA, and between daily step counts and total MVPA, in both groups (Fig. 2).

## Discussion

In this cross-sectional study, first, we investigated whether older women with LS have lower locomotive and non-locomotive MVPA compared to those without LS, and then, we examined the associations between locomotive/non-locomotive MVPA and walking ability with and without LS. The main findings of the present study were as follows: (1) daily step counts, and all PA measured by using a triaxial accelerometer were not significantly different in older women with LS compared to those without LS; (2) significant correlations were observed between locomotive MVPA and walking speed, and between total MVPA and walking speed, in the LS group, but not in the NL group; (3) a significant correlation was observed between locomotive MVPA and daily step counts, between non-locomotive MVPA and daily step counts, between total MVPA and daily step counts in both groups. These findings indicated that the amount of locomotive MVPA, non-locomotive MVPA, and total MVPA in community-dwelling older females with/without LS has maintained. In addition, there findings indicated that walking speed in older people with LS that have difficulties in locomotion is strongly influenced by locomotive MVPA the same way as old people without LS. These are out of line with our hypothesis.

Daily step counts and all PA were not significantly different between the two groups by ANCOVA adjusted for age and weight (Table 2). The values of the duration of total MVPA in two groups satisfied the MVPA value recommended by the “Active Guide” for improving physical function (i.e., 23 METs-h/week, corresponding to 3.2 METs-h/day [6]). In the NL group, the duration of locomotive MVPA was similar to, and that of non-locomotive MVPA shorter than, those reported previously by Okumatsu et al. [24] (2.0 ± 1.9 METs-h/day and 2.5 ± 2.3 METs-h/day, respectively) in middle-aged and old participants (17 females aged 54.1 ± 6.6 years and 19 males aged 68.0 ± 6.1 years) [24]. Oshima et al. [9] also reported a comparable duration of non-locomotive MVPA (14.3 ± 9.2 METs-h/weeks, corresponding to 2.0 METs-h/day) in community-dwelling Japanese older people (50 females, aged 65.0 ± 4.1 years), which was similar to those of the LS and NL groups in the present study; the duration of locomotive MVPA, on the other hand, was 11.8 ± 6.8 METs-h/weeks (1.6 METs-h/day). Considering the age-related decline in MVPA, the amount of non-locomotive MVPA and rate of non-locomotive MVPA for total MVPA in both groups has attained the value of the previous reports [10], but only locomotive MVPA in the LS group was lower than the previously reported value. Therefore, these results indicate that locomotive MVPA in the LS group has limited, but non-locomotive MVPA has been maintained. Iwaya et al. [25] reported that activity limitation due to LS may occur in the following order: sports activities, walking, transportation, and self-care [25]. The present study showed that the decline in the locomotive MVPA in the LS group was consistent with the previous study.

A significant correlation was observed between locomotive MVPA and walking speed in the LS group (*r* = 0.293, *p* < 0.05), but not in the NL group (*r* = 0.183, *p* = 0.09). Previous studies have shown that the duration of MVPA affects walking speed [12], and that walking speed is a predictor of ADL impairment [18] and independence in ADL [26, 27]. The results of the present study in LS groups were consistent with the previous findings that the slow walking speed was concerned with the lower in total MVPA. A significant correlation was observed between locomotive MVPA and walking speed in both groups. These results suggested that, for older people with LS, the duration of locomotive MVPA may be more important than that of non-locomotive MVPA for achieving a sufficient walking speed the same way as old people without LS .

Daily step counts were significantly correlated with locomotive MVPA in both groups (NL *r* = 0.736, *p* < 0.05; LS: *r* = 0.772, *p* < 0.05), and significantly correlated with non-locomotive MVPA in the both groups (*r* = 0.396; *p* < 0.001, LS *r* = 0.256, *p* = 0.092). Osuka et al. found a correlation between MVPA and ADL in community-dwelling Japanese older people, reporting that the quality of daily living and limited range of activity influenced MVPA, and that non-locomotive MVPA increased with daily step counts [28]. These previous findings are consistent with our results concerning the both groups. The 2-step test in the LS group was significantly correlated with locomotive MVPA (*r* = 0.282, *p* < 0.05), whereas the 2-step test in the NL group was significantly correlated with non-locomotive MVPA (*r* = 0.216, *p* < 0.05). The walking limitation may occur most early in ADL limitation in old people with/without LS [25, 29]. In addition, Shimada et al. reported that a physically active lifestyle is related to walking ability, balance function, and ADL and that physically active ADL such as household work is required to maintain physical function such as walking ability, balance function [30]. Therefore, these results of the 2-step test in this study indicate that the 2-step test in the LS group that walking has limited was associated with locomotive MVPA due to declining balance function and lower walking ability. On the other hand, the 2-step test in the NL group was associated with non-locomotive MVPA such as household work because of maintaining the walking ability and balance function. A significant correlation between locomotive MVPA and daily step counts may be indicated that daily step counts associate with locomotive MVPA even if older people with LS have difficulties in locomotion. Thus, while increasing step counts may be effective in achieving MVPA that is required to improve and maintain physical function in the LS group the same way as old people without LS.

Daily step counts were not significantly different in two groups (LS group 6024 ± 2408 steps/day, NL group 6994 ± 2417 steps/day). Tudor-Locke et al. [31] systematically reviewed the relationship between MVPA and step counts, and reported that 30 min of MVPA, which corresponds to roughly 7000–10,000 steps/day, is required to maintain and improve physical function and a physically active lifestyle. Most of the participants in the NL group met this requirement, but some in the LS group did not achieve this. Shinkai et al. [32] reported that mobility declines with decreasing PA in older people, even if their walking ability remains high.

This study has several limitations. First, the present study included the participants who had voluntarily participated in the community health-promoting events and were thus health-conscious. Second, the PA data may potentially be biased, since even though participants were instructed to spend an ordinary life, their daily step counts might have increased as a result of wearing the accelerometer (i.e., increased health consciousness). Third, since the present study adopted a cross-sectional design, the results should be validated in a prospective study with a larger sample size. Finally, in the previous study, the methods to distinguish locomotive activity and non-locomotive activity for older by using the accelerometer have been applied to the healthy participants [10, 33]. The validity of the accelerometer for the subjects with LS needs to be verified in the future study.

## Conclusions

Walking speed was positively correlated with locomotive MVPA in the LS group. This result suggests that slow walking speed in older people with LS occurs in connection with lower locomotive MVPA. These findings may contribute to the treatment of older females with LS.

## Data Availability

The datasets used and/or analyzed during the current study are available from the corresponding author on reasonable request.

## Abbreviations

LS: Locomotive syndrome
MVPA: Moderate to vigorous physical activity
NL: Normal
MET: Metabolic equivalent tasks
JOA: Japanese Orthopedics Association
ADL: Activities of daily living
GLFS-25: Question Geriatric Locomotive Function Scale
MHLW: Ministry of Health, Labour and Welfare
BMI: Body mass index
AMM: Appendicular muscle mass
SMI: Skeletal muscle index
BIA: Bioelectrical impedance analysis
TMIG-IC: Tokyo Metropolitan Institute of Gerontology, Index of Competence
LPA: Low-intensity physical activity
SD: Standard deviation
